# Characteristics and Role of Neutrophil Extracellular Traps in Asthma

**DOI:** 10.1007/s10753-021-01526-8

**Published:** 2021-09-04

**Authors:** Fei Chen, Min Yu, Yonghong Zhong, Lina Wang, Huaqiong Huang

**Affiliations:** 1 Key Laboratory of Respiratory Disease of Zhejiang Province, Department of Respiratory and Critical Care Medicine, Second Affiliated Hospital of Zhejiang University School of Medicine, Hangzhou, Zhejiang China; 2 Linping Campus, The Second Affiliated Hospital of Zhejiang University School of Medicine, Hangzhou, Zhejiang China

**Keywords:** Neutrophil, Neutrophil extracellular traps, Asthma.

## Abstract

Asthma is a common chronic respiratory disease that affects millions of people worldwide. The incidence of asthma has continued to increase every year. Bronchial asthma involves a variety of cells, including airway inflammatory cells, structural cells, and neutrophils, which have gained more attention because they secrete substances that play an important role in the occurrence and development of asthma. Neutrophil extracellular traps (NETs) are mesh-like structures composed of DNA, histones, and non-histone molecules that can be secreted from neutrophils. NETs can enrich anti-bacterial substances and limit pathogen migration, thus having a protective effect in case of inflammation. However, despite of their anti-inflammatory properties, NETs have been shown to trigger allergic asthma and worsen asthma progression. Here, we provide a systematic review of the roles of NETs in asthma.

## INTRODUCTION

Neutrophils are terminally differentiated white blood cells which have a survival span of 6–8 h in circulation, but can be long-lived after infiltrating tissues [1–3]. Inflammatory stimuli induce neutrophils to leave the circulation and migrate to the site of infection where they play a critical role in phagocytosis, degranulation, and reactive oxygen species (ROS) production. Neutrophils can also release neutrophil extracellular traps (NETs) that degrade virulence factors, as well as engulf and kill pathogens [4–6]. NETs have also been shown to have a role in autoimmune diseases [7–9], chronic airway inflammatory diseases [10, 11], and thrombotic diseases [12].

Asthma is a common chronic airway inflammatory disease that affects people of all races, ethnicities, and ages. According to Chinese asthma guidelines (2020), patients were divided into mild, moderate, and severe asthma according to the frequency of symptoms, the effect on activity and sleep, and the change of lung function [13]. NETs components such as antimicrobial LL-37, α-defensins 1–3, and neutrophil elastase(NE) were significantly elevated in asthma compared with healthy controls [14]. Interestingly, NETs increased with disease progression [14, 15]. Mouse models of asthma have indicated that an elevation in NETs secretion leads to increased cell infiltration, mucus secretion, and airway inflammation [16].

Although it remains unknown how NETs contribute to the pathogenesis of chronic inflammatory airway diseases, the importance of NETs should not be overlooked. Understanding the mechanisms by which NETs are involved in inflammatory diseases can provide the foundation for developing new diagnostic tools and effective treatments of chronic inflammation and autoimmune diseases [17]. This review summarizes the current knowledge on the general characteristics of NETs, their anti-microbial properties, and their role in the development of asthma.

## NEUTROPHILS AND NETS

Neutrophils account for approximately 70% of the leukocyte population in the peripheral blood and are considered the first line of defense against pathogens in the innate immune system [18]. About 50–100 billion neutrophils are generated in the bone marrow, and one billion are released into the circulation [19]. Most neutrophils entering the circulation are mature phenotype. Neutrophils exist as two subtypes after being stimulated by allergens and pathogens: immature or band nuclear neutrophils and hypersegmented neutrophils. Hypersegmented neutrophils have a longer lifespan and can form NETs and produce myeloperoxidase (MPO) and matrix metalloproteinase (MMP) [2, 20, 21]. The prolonged lifespan of these neutrophils allows these cells to migrate back from the tissues to the lymph nodes or circulation to mediate an adaptive immune response. Therefore, it is necessary to further explore and understand the different subtypes of neutrophils, as well as the molecules they secrete, to clarify their specific role in the pathogenesis of asthma in the future.

## STRUCTURE OF NETS

Upon activation, neutrophils may undergo a specific type of cell death called NETosis, which is characterized by the release of NETs and is distinctly different from necrosis and apoptosis [22, 23]. NETs are composed of decondensed chromatin and granules/protein, including NE, MPO, cathepsin G, α-defensins 1–3, and high-mobility group box 1 protein (HMGB1) [4, 17, 24]. However, the full protein composition is not completely clear. Zychlinsky and colleagues reported 24 NETs-associated proteins including nuclear, granular, and cytoplasmic proteins. Among these, histones accounted for 70% of all NETs-associated proteins, but non-histone NE was the most abundant and catalase was the least abundant [25].

## NETS FORMATION

A variety of factors can induce NETs formation, such as lipopolysaccharide (LPS), bacteria, viruses, intracellular molecules (i.e., interleukin (IL)-8, ROS, IL-37), and chemicals (i.e., phorbol 12-myristate 13-acetate (PMA)) [23, 26]. It has been shown that PMA is the strongest inducer of NETs, which also strongly induces ROS generation in vitro [17]. NETs formation can be NADPH enzyme-dependent or independent, and the formation process can be divided into cell membrane rupture and non-rupture.

### Suicidal NETosis

Fuchs and colleagues reported that after PMA stimulation, neutrophil nuclei begin to lose lobules, chromatin decondenses, and the gap between the inner and outer nuclear membranes expands, but the nuclear membrane remains intact. However, after 1 h of stimulation, the nuclear membrane disintegrates into vesicles and the granular membrane disappears, allowing the mixing of chromatin and granular components. Once the nucleoplasm and cytoplasm mix, the cell membrane ruptures, allowing NETs to be extruded, which occurs approximately 3–4 h after simulation [27]. NETs formation is the last step in the process of cell death, and NETs are released at the moment of activated neutrophil death [27].

At the molecular level, activation of NADPH and the Raf-MEK-ERK pathway is involved in the formation of suicidal NETs [28]. Diphenylene iodonium (DPI), a NADPH oxidase inhibitor, decreases NETs formation and ROS production upon PMA activation [27]. In lupus-prone mice deficient in the NADPH oxidase, neutrophils cannot make NETs and the mice have worsened lupus [29]. Interestingly, neutrophils isolated from patients with chronic granulomatous disease, which is characterized by mutations in NADPH oxidase, cannot form NETs either [27]. Activation of the NADPH oxidase (Nox2)results in ROS generation and disintegration of the nuclear membrane and most granule membranes, leading to chromatin decondensation and NETs formation [30]. Nitric oxide synthase (NOS) and MPO can also produce ROS and contribute to NETs formation [15, 31].

The cytokine IL-8 forms NETs through suicidal or terminal NETosis [32], but the mechanism by which IL-8 forms NETs is unclear. IL-8 can bind to two different receptors: IL-8 receptor alpha (CXCR1) and IL-8 receptor beta (CXCR2) [33]. Marcos et al*.* noted that IL-8 forms NETs through a CXCR2-mediated NADPH oxidase independent mechanism in pulmonary cystic fibrosis [34]. Activation of CXCR2 does not affect NADPH or the production of ROS, while CXCR1 is involved in oxidative stress and phospholipase D (PLD) activation. Phosphatidic acid (PA), the direct product of PLD, is considered to be an activator of NADPH oxidase, so the formation of NETs may also be related to CXCR1 [35].

### Vital NETosis

Suicidal NETosis requires membrane rupture, resulting in bacteria escape and the loss of conventional neutrophil functions, such as recruitment, chemotaxis, and phagocytosis. Several groups have proposed a novel mechanism of NETosis that is not dependent on neutrophil lysis [36, 37]. Specifically, *Staphylococcus aureus* infection results in nucleus rounding and rapid condensing, separation of the inner and outer nuclear membranes, and vesicle budding, while the plasma membrane remains intact. The budding vesicles extend into the extracellular space and rupture, releasing chromatin, and some cytoplasmic particles are released into the extracellular space to form NETs [36]. This form of NETosis is oxidant-independent and happens more quickly than suicidal NETosis, which occurs in 5–60 min. With time, the nuclear membrane breaks and DNA fills the cytoplasm, which prolongs NETosis [36]. This mechanism appears to be triggered by LPS and other microorganisms which activate the Toll-like receptor (TLR) [26] in an early/rapid ROS-independent pathway that does not affect neutrophil viability [38]. Platelet's detection of TLR4 ligands (such as LPS) can induce platelet-neutrophil interaction, leading to neutrophil activation and NETs formation [37]. However, other groups have suggested that NETs formation is not dependent on platelet-TLR interaction [39].

## COMMON NETOSIS PROCEDURES

In addition to nuclear membrane and plasma membrane’s change, NETs formation requires chromatin decondensation. Peptidyl arginine deiminase 4 (PAD4), NE, and MPO are related to the formation and release of NETs [40, 41]. PAD4 is responsible for histone hypercitrullination-induced chromatin decondensation in neutrophils [40, 42]. Following PAD4 inhibition or in vivo knockout of PAD4 (PAD4 − / −) in mice, neutrophils form fewer NETs compared to wild-type mice [16, 43]. NE translocates from cytoplasm granules to the nucleus and degrades specific histones, promoting chromatin decondensation. Subsequently, MPO synergizes with NE [41], but these enzymes have different roles in different NETs formation types. One study showed that *Leishmania* parasites can induce suicidal and vital NETosis. The ROS formation, PAD4, and elastase were involved in suicide NETosis, while elastase, but not ROS or PAD4, were involved in vital NETosis [44]. Although numerous studies have focused on NETs, the mechanisms underlying NETs formation, including stimulating factors, molecular pathways, and the formation process, are still not fully understood.

## FUNCTIONS OF NETS

### Protective Functions

NETs are part of the innate immune response, which helps to capture Gram-positive bacteria, Gram-negative bacteria [4, 39], fungi [25], viruses [45], and parasites [46]. The structural integrity of NETs is critical for killing bacteria; if NETs are degraded by DNase, their anti-microbial power is significantly reduced [4]. NE degrades virulence factors of Gram-negative bacteria [4], and serine protease can penetrate and destroy bacterial membranes [47]. Other NETs components, such as histones, anti-microbial peptides, lysozymes, and defensins, play a synergistic anti-microbial role [1, 4]. However, there is growing evidence demonstrating that many microorganisms, such as Group A *Streptococcus* and *Streptococcus pneumoniae*, can avoid being captured by NETs by synthesizing DNA enzymes, resulting in immune escape [48, 49].

### Harmful Functions

It is known that excessive production and inefficient degradation of NETs may cause inflammation and tissue damage. A study has shown that extracellular histones were highly pro-inflammatory and caused serious pulmonary damage [50]. Another study indicated that histones, which are the primary component in NETs, mediate epithelial and endothelial cell damage as a result of lactate dehydrogenase (LDH) spillover and an increase in apoptotic protein activity [51] that influence barrier integrity to make allergen stimulation augment. Marrah and Hudock et al*.* demonstrated that NETs could stimulate IL-1, IL-6, and IL-8 cytokine secretion in human airway epithelia [52, 53] which may contribute to neutrophil recruitment and aggravate airway inflammation in asthma.

In addition to epithelial cells, NETs can act on antigen presenting cells (DCs) and contribute to autoimmune diseases, such as systematic lupus erythematosus and psoriasis [54, 55]. As researches showed, NETs increased cytokines and chemokine secretion like IL-6, TNF-α, and GM-CSF which function in DCs maturation [56]. NETs significantly increased surface expression of co-stimulatory molecules (CD40, CD80, CD86) on DCs combined with cytokine secretion of IFN-α, IL-6, and IL-12/p70 and further differentiated CD4 + T cells into various subtypes like Th1 and Th17 cells in cigarette-smoke exposed mice, resulting in airway inflammation and promoting asthma development [20, 57]. When DCs were directly stimulated with NETs, the co-stimulatory molecules were elevated, and DC-derived IL-6 and TNF-α secretion were enhanced as well. In general, NETs can recruit and activate DCs, increase the expression of surface co-stimulatory molecules, and promote DCs to present antigens in adaptive immune response that bridged with the innate immune response in chronic inflammation [20].

## NETS AND ASTHMA

Asthma is a chronic heterogeneous airway inflammatory disease that affects approximately 300 million people worldwide. Asthma is characterized by airway inflammation, reversible airflow obstruction, and airway hyperresponsiveness [58–60]. Asthma symptoms include recurrent wheezing, coughing, and shortness of breath [61]. According to the *Chinese Asthma Guideline 2020*, asthma can be classified into mild, moderate, and severe asthma. Based on the treatment response, asthma can also be categorized as glucocorticoid sensitive or insensitive. Previous studies have shown that asthma is mainly driven by Th2 cells secreting IL-4, IL-5, and IL-13, leading to airway eosinophil inflammation [62]. With further research, however, it has been found that asthma can be caused by airway neutrophilic inflammation, especially in acute, severe, and glucocorticoid insensitive asthma [63]. This classification is based on the proportion of eosinophils and neutrophils in induced sputum. In summary, asthma is classified into either eosinophilic, neutrophilic, mixed granulocytic, or paucigranulocytic according to the type of infiltrating cell in the induced sputum.

### Role of NETs in Early Asthma

Neutrophils and its secretion, NETs, play a vital role in the pathogenesis of asthma. Jancar et al. pointed out that after ovalbumin (OVA) stimulation in mice, neutrophils in the bronchial alveolar lavage fluid (BALF) increased at 6 h, peaked at 12 h, and then decreased, while eosinophils increased at 18 h [64]. Toussaint et al. showed that neutrophils increased significantly on the first day and NETs formed after rhinovirus (RSV) stimulation in a murine asthma model, involved in immune responses and asthma pathology, but there was no significant change in eosinophils. [65]. A more recent study has shown that in an LPS- and house dust mite (HDM)-induced asthma model, a population of neutrophils that specifically expresses CXCR4 were recruited early in the airway response, which release NETs. NETs derived from CXCR4 neutrophils were also needed to mediate allergic asthma triggered by infection with influenza virus or exposure to ozone [16]. In addition, studies showed that neutrophils increased more earlier at 3 h after an initial OVA stimulation releasing of neutrophil elastase—a major component of NETs, while eosinophils began to rise at 24 h. Weng et al. further investigated the role of macrophages in the inflammatory response. They showed that macrophages could induce neutrophil infiltration by producing chemokines following allergen stimulation [66]. These studies suggest that neutrophils are involved in early asthma, while eosinophils play a role in late asthma responses.

Pham et al. showed that NETs formation peaked on the second day post-Sendai virus infection and that NETs played a crucial role in the early immune response by recruiting and activating leukocytes such as CD4 + and CD8 + T cells and increasing TNF-α, IL-6, and pathogenesis of asthma through increased TNF-α aggravates AHR as well as recruits inflammatory cells with a positive feedback [67]. This effect was confirmed by other researchers. Marichal et al*.* showed that NETs can promote the presentation of antigen by DCs and induce a Th2 inflammatory response in asthma mice model induced by LPS-HDM, as evidenced by increased secretion of Th2 cytokines, eosinophil infiltration, mucus hypersecretion, and airway hyperresponsiveness [16]. These results indicate that the early infiltration of neutrophils and the formation of NETs are involved in the early stage of asthma.

### Role of NETs in Asthma Progression

The role of NETs in asthma progression has been debated. The neutrophil population has been shown to increase in induced sputum or BALF samples from patients with severe asthma, acute exacerbation of asthma, and persistent asthma [68–70]. In children with acute asthma, the level of NETs in the peripheral blood were also increased [15]. Extracellular DNA (eDNA), a main component of NETs, was detected at a higher level in sputum samples from those with severe asthma compared to those with mild/moderate asthma [14]. However, as shown in another study, NETs increased in the circulation but not in the BALF [71]. Neutrophils and NETs were not shown to increase after inhalation of allergens in airway biopsy specimens [72], and the effects on NETs in the peripheral blood remain unclear. We suspect BALF may not well represent airway NETs levels than sputum, and circulating NETs levels, which reflect neutrophil activation, could become another useful marker of asthma severity and poor control [71]. However, to what degree NETs levels in the peripheral blood or airway change in severe asthma and acute exacerbation asthma remain unknown, as do the factors that are involved in NETs fluctuation. Thus, further clinical research is needed to understand the role of NETs in asthma progression.

The exact mechanism underlying asthma progression has not been clarified, but evidence suggests that airway epithelium disruption is a primary cause. Virus, other microorganisms, and allergen stimulation will make increased neutrophil recruitment. Neutrophil can produce NETs by vital NETosis through directly microbial stimulation or suicidal NETosis with elevated IL-8 secreted by leukocytes. Increased NETs can disrupt bronchial epithelial tight junctions, which leads to intracellular component spillover [73], and NETs can directly act on airway epithelial cells to secrete inflammatory factors, aggravating airway inflammatory and increasing respiratory symptoms [52, 74]. HMBG1, a NETs component, can activate bronchial epithelial cells and increase the expression of thymic stromal lymphoprotein (TSLP), tumor necrosis factor-α (TNF-α), MMP-9, and vascular endothelial growth factor (VEGF) and its related p38 MAPK and ERK1/2 signaling pathways [75]. Protease, which accounts for 10% of NETs content, has also been shown to activate cytokines, including IL-1, IL-33, and IL-36, thus aggravating the inflammatory response [76]. Clinical data from nasopharyngeal samples show that children with RSV infection have a large amount of neutrophil infiltration, accompanied by a significant increase in TSLP, IL-33, IL-10, and periostin. With severe infection, the levels of TSLP, IL-33, and IL-10 are even higher [77]. Therefore, we propose that NETs destroy airway epithelium integrity, increase cytokine secretion, and lead to asthma progression.

## NETS AND THERAPY

Antagonists targeting NETs components can reduce airway inflammation in asthma [65]. In murine experiments, the use of NETs synthesis inhibitors or NET degradation agents (PAD4 inhibitors, elastase inhibitors, and DNA enzymes) have been shown to reduce NETs formation, leading to a decrease in inflammatory cell infiltration, inflammatory score, goblet cell proliferation, and mucus secretion, as well as significantly reduce airway resistance in vivo [16, 78]. Dornase alfa is an approved atomized recombinant human deoxyribonuclease (rhDNase) used in cystic fibrosis (CF). It reduces fluid viscosity in a dose-dependent manner, and long-term use can improve lung function in CF patients [79]. In autoimmune diseases, such as SLE and granulomatosis with polyangiitis (GPA), neutrophils form NETs more frequently, but their ability to degrade NETs is decreased [80]. In asthma, airway mucus emboli can be achieved within minutes after the administration of a recombinant human DNA enzyme [81]. Therefore, either controlling NETs formation or promoting NETs degradation may be an effective strategy for treating asthma.

## CONCLUSION

Brinkmann et al. were the first to propose NETs (4). Since then, new mechanisms of neutrophil-mediated killing of pathogens has enriched our understanding of neutrophil function. NETs can prevent the spread of bacteria and limit local inflammation, but can also cause tissue damage and aggravate disease long-term. NETs are not only involved in the pathogenesis of asthma, but are also involved in many other autoimmune and inflammatory diseases, including chronic obstructive lung disease, sepsis, and vascular diseases. Although numerous studies have investigated NETs formation and function, the specific mechanisms by which NETs are regulated remain unknown. A further understanding of the molecular mechanisms underlying NETs formation and function would provide new insights into the pathogenesis and treatment of asthma.

## Data Availability

Not applicable.
